# Systemic modulation of stress and immune parameters in patients treated for prostate adenocarcinoma by intensity-modulated radiation therapy or stereotactic ablative body radiotherapy

**DOI:** 10.1007/s00066-020-01637-5

**Published:** 2020-06-09

**Authors:** B. Frey, J. Mika, K. Jelonek, L. Cruz-Garcia, C. Roelants, I. Testard, N. Cherradi, K. Lumniczky, S. Polozov, A. Napieralska, P. Widlak, U.S. Gaipl, C. Badie, J. Polanska, S. M. Candéias

**Affiliations:** 1 Department of Radiation Oncology, Universitätsklinikum Erlangen, Friedrich-Alexander-Universität Erlangen-Nürnberg, 91054 Erlangen, Bavaria, Germany; 2 Department of Data Science and Engineering, Silesian University of Technology, 44-100 Gliwice, Poland; 3 Maria Sklodowska-Curie National Research Institute of Oncology, Gliwice Branch, 44-102 Gliwice, Poland; 4 Centre for Radiation, Chemical and Environmental Hazards, Cancers Mechanisms and Biomarkers group, Public Health England, Chilton, OX11 ORQ Didcot, Oxfordshire UK; 5 Inovarion, 75005 Paris, France; 6 Univ. Grenoble Alpes, CEA, CNRS, IRIG-LCBM-UMR5249, 38054 Grenoble, France; 7 Univ. Grenoble Alpes, INSERM, CEA, IRIG-BCI-UMR_S1036, 38054 Grenoble, France; 8 National Public Health Center, 1097 Budapest, Hungary; 9 HQ Science Limited, 5 The Quay, PE27 5AR St. Ives, Cambridgeshire, United Kingdom

**Keywords:** Ionizing radiation, Biomarkers of radiation exposure, Prostate cancer, Systemic immune modulation, Immunophenotyping

## Abstract

**Background:**

In this exploratory study, the impact of local irradiation on systemic changes in stress and immune parameters was investigated in eight patients treated with intensity-modulated radiation therapy (IMRT) or stereotactic ablative body radiotherapy (SABR) for prostate adenocarcinoma to gain deeper insights into how radiotherapy (RT) modulates the immune system.

**Patients and methods:**

RT-qPCR, flow cytometry, metabolomics, and antibody arrays were used to monitor a panel of stress- and immune-related parameters before RT, after the first fraction (SABR) or the first week of treatment (IMRT), after the last fraction, and 3 weeks later in the blood of IMRT (*N* = 4) or SABR (*N* = 4) patients. Effect size analysis was used for comparison of results at different timepoints.

**Results:**

Several parameters were found to be differentially modulated in IMRT and SABR patients: the expression of *TGFB1*, *IL1B*, and *CCL3* genes; the expression of HLA-DR on circulating monocytes; the abundance and ratio of phosphatidylcholine and lysophosphatidylcholine metabolites in plasma. More immune modulators in plasma were modulated during IMRT than SABR, with only two common proteins, namely GDF-15 and Tim‑3.

**Conclusion:**

Locally delivered RT induces systemic modulation of the immune system in prostate adenocarcinoma patients. IMRT and SABR appear to specifically affect distinct immune components.

**Electronic supplementary material:**

The online version of this article (10.1007/s00066-020-01637-5) contains supplementary material, which is available to authorized users.

## Background

Approximately 50% of all cancer patients will receive radiotherapy (RT) during their course of illness. RT aims to kill tumor cells while sparing as much as possible of the surrounding healthy tissues [1]. Intensity-modulated radiation therapy (IMRT) and stereotactic ablative body radiotherapy (SABR) are used to perform a more precise irradiation of the tumor volume [2]. While radiation delivery during IMRT is conventionally fractionated (e.g., 2 Gy/fraction, 5 days/week), it is hypofractionated during SABR (e.g., 7.25 Gy/fraction, 5 fractions every second day). Thus, widely different RT protocols are nowadays used for the treatment of similar tumor entities. RT has long been regarded as local therapy, but this point of view changed when data on systemic effects of locally delivered radiation were collected [3]. Biomarkers of radiation exposure have been discovered [4] in the blood of patients after local [5, 6] or total body [7–9] irradiation. These bioindicators include genes such as *FDRX* (coding for ferredoxin reductase, a mitochondrial protein involved in electron transport), *DDB2* (coding for the damage-specific DNA binding protein 2, involved in DNA repair), *MDM2* (coding for the mouse double minute 2 proto-oncogene, involved in the regulation of p53 degradation), and *SESN1, GADD45*, and *CCNG1* (coding, respectively, for the sestrin 1 protein, the growth arrest and DNA damage-inducible protein 45, and the cyclin G1 protein, involved in the inhibition of cell cycle and growth arrest in stressful conditions). Expression of these genes is induced by p53 after activation of the radiation-induced DNA damage response checkpoints. Interestingly, the expression of some inflammatory genes such as *ARG1* (coding for arginase 1, involved in amino acid metabolism and cell proliferation), *BCL2L1 *(coding for a protein of the BCL2 family, involved in the control of apoptosis), and *MYC* (coding for the MYC proto-oncogene, involved in cell cycle progression, apoptosis, and cellular transformation) is also found to be dysregulated [8]. While expression of *ARG1* and *BCL2L1* was upregulated, *MYC* expression was decreased in the blood of cancer patients during RT. These findings show that RT can result in long-term modification of gene expression and potential immunomodulatory effects [3]. In fact, tumor development is intimately linked to the negative modulation of immune cell functions [10]. Tumor cells can, for example, up-regulate their expression of immune checkpoint molecules such as programmed death ligand 1 (PD-L1), making them, e.g., less susceptible to killing by cytotoxic T lymphocytes. It has been proposed that the anti-tumor efficiency of RT can benefit from a synergy of concomitant re-activation of the immune system [11–13].

Systemic effects of ionizing radiation could also be observed at the level of the proteome, including cytokines, and metabolome in the blood of cancer patients treated with RT [14]. Early changes in the plasma level of a collection of cytokines were, for example, associated with toxicity in patients treated for lung cancer, and this response was modified by the combination of chemotherapy with RT [15]. The intensity of radiation-induced toxicity, including the inflammatory response, is one key factor affecting RT-related changes in the blood proteome [16]. RT-related changes could be also detected in the serum lipidome and dynamic radiation-induced changes in the levels of phosphatidylcholines (PCs) and lysophosphatidylcholines (LPCs) were observed in patients treated for head and neck cancer [17]. LPCs are pro-inflammatory lipids involved in atherosclerosis [18] and an increased plasma LPC/PC ratio has been reported in certain pro-inflammatory conditions [19]. It was recently reported that the LPC/PC ratio increased in the serum of whole-body-irradiated mice [20]. Hence, this parameter represents a potential inflammation-related metabolomic marker of the response to radiation.

However, only few data exist on joint analyses of the manifold stress and immune changes that might occur following local treatment of solid tumors such as prostate adenocarcinoma. Such information could shed further light on how a distinct RT scheme impacts at the systemic level and might help to improve multimodal therapies in the future.

In this exploratory study, we therefore complementarily analyzed the expression of stress-response and inflammatory cytokine genes, the modulation of immune cell populations, changes in metabolites, and changes in cytokines levels in the blood of a group of 8 patients undergoing RT for prostate adenocarcinoma. As a first attempt to find out whether modulation of these parameters at the systemic level depends on factors such as the irradiated volume, the dose delivered per fraction, the dose rate, and the total dose received, biomaterial of patients who were treated with two markedly different RT modalities, namely IMRT and SABR, was used for the analyses.

## Materials and methods

### Patients and RT treatment

Male patients diagnosed with prostate adenocarcinoma aged from 63 to 83 years (median 70 years) were recruited for the exploratory study. These patients had no surgery and had not received chemotherapy previously. However, some of them were subjected to androgen-deprivation therapy (ADT), as indicated in Supplementary Table 1. Four patients were treated with photon IMRT (volumetric arc therapy, VMAT; energy 6 MV, dose rate 3 Gy/min) with a daily fraction of 2 Gy, according to the conventional five times a week irradiation scheme. The total radiation dose delivered to prostate and half of seminal vesicles (clinical target volume, CTV) was 78 Gy; during the first 22 days of RT, the CTV additionally included the region of the pelvic lymph nodes (total dose of 44 Gy). Overall treatment time was in the range of 53–57 days. Another four patients were treated with SABR using a CyberKnife (Accuray Inc., Chesapeake Terrace Sunnyvale, CA, USA) treatment unit according to the scheme 5 fractions of 7.25 Gy every second day (energy 6 MV, dose rate 9 Gy/min). The total radiation dose delivered to the CTV was 36.25 Gy and overall treatment time was in the range of 9–12 days. The CTV in these patients included the prostate and base of the seminal vesicles. All recruited patients signed informed consent indicating their conscious and voluntary participation. One IMRT patient died from a non-cancer-related cause 3 months after RT completion; the seven other patients are still alive 3 years after treatment (Supplementary Table 1).

**Table 1:** Patients recruited for this study

Patient	Pathology	Age (year)	Total dose (Gy)	Number of fractions	Fraction dose (Gy)	Overall treatment time (days)	Sample B: dose (Gy)	Sample B: days after 1st fraction	Sample C: dose (Gy)	Sample C: days after last fraction	Sample D: days after last fraction
SABR‑1	Adenocarcinoma, Gleason (3 + 3)	68	36.25	5	7.25	10	7.25	2	36.25	0	34
SABR‑2	Adenocarcinoma, Gleason (3 + 3)	68	36.25	5	7.25	9	7.25	2	36.25	0	31
SABR‑3	Adenocarcinoma, Gleason (3 + 4)	75	36.25	5	7.25	12	7.25	5	36.25	0	30
SABR‑4	Adenocarcinoma, Gleason (3 + 3)	68	36.25	5	7.25	12	7.25	3	36.25	0	38
IMRT‑1	Adenocarcinoma, Gleason (3 + 3)	63	78	39	2.0	53	10.0	7	78.0	2	37
IMRT‑2	Adenocarcinoma, Gleason (4 + 4)	79	78	39	2.0	57	10.0	7	78.0	0	31
IMRT‑3	Adenocarcinoma, Gleason (4 + 3)	72	78	39	2.0	57	10.0	7	78.0	0	31
IMRT‑4	Adenocarcinoma, Gleason (4 + 4)	83	78	39	2.0	53	10.0	7	78.0	0	36

*SABR* stereotactic ablative body radiotherapy, *IMRT* intensity-modulated radiation therapy

**Fig. 1:**
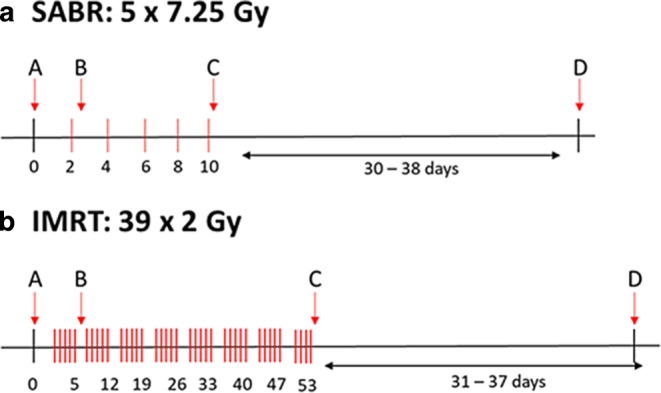
Schematic presentation of the blood collection timeline of the prostate adenocarcinoma patients treated with SABR (**a**) and IMRT (**b**). *Red lines* represent radiation fractions of 7.25 Gy and 2 Gy for SABR and IMRT, respectively. The approximative days of delivery are indicated under the time axis: for each fraction for SABR and at the end of each group of 5 fractions for IMRT. Please note the different time scale for the different RT modalities. *SABR* stereotactic ablative body radiotherapy, *IMRT* intensity-modulated radiation therapy

Whole blood of the patients was collected within a week before the start of treatment (sample A), approximately 40 h after the fifth IMRT fraction (cumulative dose 10 Gy) or after the first SABR fraction (sample B), within 6 h after the last fraction (sample C), and approximately 5 weeks after the last fraction (sample D). A schematic timeline of blood collection is presented in Fig. 1. The blood collection times and doses are specified for each patient in Table 1, while a full description of the patients, their pathology, and the treatment is provided in Supplementary Table 1. This study was carried out in accordance with the Bioethical Committee in the Maria Sklodowska-Curie Institute, Warszawa, approval number 27/2015 from 18/08/2015.

### RNA isolation and reverse transcription

Total RNA from samples collected in PAXgene tubes from RT patients was extracted with the PAXgene Blood miRNA kit (Qiagen, PreAnalytiX GmbH, Hilden, Germany) using a robotic workstation Qiacube (Qiagen, Manchester, UK). The quantity of isolated RNA was determined by spectrophotometry with a ND-1000 NanoDrop and quality was assessed using a Tapestation 220 (Agilent Technologies, Santa Clara, CA, USA). cDNA was prepared from 350 ng of total RNA using the High Capacity cDNA reverse transcription kit (Applied Biosystems, FosterCity, CA, USA) according to the manufacturer’s protocol. Alternatively, total RNA was reverse-transcribed using the HS-RT100 kit (Sigma-Aldrich, L’Isle d’Abeau, France) with anchored oligo dT priming according to the manufacturer’s protocol.

### Quantitative real-time polymerase chain reaction

Two different quantitative PCR (qPCR) protocols were used. First, qPCR was performed using a Rotor-Gene Q (Qiagen, Hilden, Germany) with PerfeCTa MultiPlex qPCR SuperMix (Quanta Bioscience, Inc., Gaithersburg, MD, USA). The samples were run in triplicate in 10 µl reactions with 1 µl of the cDNA synthesis reaction together with six different sets of primers and fluorescent probes at 300 nM concentration each. 3’6-carboxyfluorescein (FAM), 6‑hexachlorofluorescein (HEX), Atto 680, Atto 390, Texas Red (Eurogentec Ltd., Fawley, Hampshire, UK), and CY5 (Sigma-Aldrich, Poole, Dorset, UK) were used as fluorochrome reporters for the probes analyzed in multiplexed reactions with six genes per run including a housekeeping gene. The reactions were performed with the following cycling conditions: 2 min at 95 °C, then 45 cycles of 10 s at 95 °C and 60 s at 60 °C. Data were collected and analyzed by Rotor-Gene Q Series Software. Gene target Ct (cycle threshold) values were normalized to the *HPRT1 *internal control. Ct values were converted to transcript quantity using standard curves obtained by serial dilution of PCR-amplified DNA fragments of each gene. The linear dynamic range of the standard curves covering six orders of magnitude (serial dilution from 3.2 × 10^−4^ to 8.2 × 10^−10^) gave PCR efficiencies between 93 and 103% for each gene with R^2^ > 0.998.

In a second set of experiments, the expression of *DDB2*, cyclin G1 (*CCNG1*), the C‑C motif chemokine ligand 3 (*CCL3*), the interleukins (IL)-1β (*IL1B*), *IL6*, *IL8*, and tumor growth factor β1 (*TGFB1*) was analyzed with a different qPCR procedure, namely SyBr Green-based real-time PCR assay. For these genes, amplification was performed in a C100 Thermal Cycler (BioRad, Marnes-la-Coquette, France) equipped with a CFX 384 Real-Time System. Samples were run in triplicate 10 µL reactions containing 5 µL of LuminoCT SYBR Green qPCR ReadyMix (Sigma-Aldrich), 2 µM of each primer, and 2 µL of 1/8th-diluted cDNA. After 2 min of denaturation at 95 °C, reactions were performed for 40 cycles consisting of 5 s of denaturation at 95 °C and 20 s of elongation at 60 °C. A melting curve was established at the end of the run to verify the amplification of a unique product in each well. Amplification curves were analyzed with the CFX Manager 3.1 software (BioRad). Cq for each PCR product was determined using the regression mode. Expression of each gene, corrected for primer efficiency, was normalized to the expression of *HPRT* and *36B4* housekeeping genes amplified concurrently on the same plate [21]. PCR runs were validated when amplification of these genes resulted in CV < 0.25 and M < 0.5 values. The efficiency of the different primer pairs was between 87 and 105%. The sequence of primers is provided in Table 2.

**Table 2:** Oligonucleotide primers used in this study

Oligonucleotide primers used in multiplex TaqMan assay	
*HPRT1*	F: 5’ TCAGGCAGTATAATCCAAAGATGGT 3’ R: 5’ AGTCTGGCTTATATCCAACACTTCG 3’ probe: 5’ CGCAAGCTTGCTGGTGAAAAGGACCC 3’
*DDB2*	F: 5’ GTCACTTCCAGCACCTCACA 3’ R: 5’ ACGTCGATCGTCCTCAATTC 3’ probe: 5’ AGCCTGGCATCCTCGCTACAACC’ 3’
*GADD45*	F: 5’ CTGCGAGAACGACATCAAC 3’ R: 5’ AGCGTCGGTCTCCAAGAG 3’ probe: 5’ ATCCTGCGCGTCAGCAACCCG 3’
*SESN1*	F: 5’ GCTGTCTTGTGCATTACTTGTG 3’ R: CTGCGCAGCAGTCTACAG 3’ probe: 5’ ACATGTCCCACAACTTTGGTGCTGG 3’
*FDXR*	F: 5’ GTACAACGGGCTTCCTGAGA3’ R: 5’ CTCAGGTGGGGTCAGTAGGA 3’ probe: 5’ CGGGCCACGTCCAGAGCCA 3’
*MDM2*	F: 5’ CCATGATCTACAGGAACTTGGTAGTA 3’ R:5’ ACACCTGTTCTCACTCACAGATG 3’ probe: 5’ CAATCAGCAGGAATCATCGGACTCAG 3’
Oligonucleotide primers used in SYBR Green assay	
*HRPT1*	S: 5’ ATGGACAGGACTGAACGTCTTGCT 3’ R: 5’ TTGAGCACACAGAGGGCTACAATG 3’
*36B4*	S: 5’ GAAATCCTGGGTGTCCGCAATGTT 3’ R: 5’ AGACAAGGCCAGGACTCGTTTGTA 3’
*CCNG1*	F: 5’ GGAGCTGCAGTCTCTGTCAAG 3’ R: 5’ TGACATCTAGACTCCTGTTCCAA 3’
*DDB2*	F: 5’ GTCACTTCCAGCACCTCACA 3’ R: 5’ ACGTCGATCGTCCTCAATTC 3’
*IL1B*	F: 5’ CTCGCCAGTGAAATGATGGCT 3’ R: 5’ GTCGGAGATTCGTAGCTGGAT 3’
*IL6*	F: 5’ CCTCGACGGCATCTCAGCCCT 3’ R: 5’ TCTGCCAGTGCCTCTTTGCTGC 3’
*IL8*	F: 5’ TGGCAGCCTTCCTGATTTCT 3’ R: 5’ ATTTCTGTGTTGGCGCAGTG 3’
*CCL*3	F: 5’ GACCGCCTGCTGCTTCAGCTA 3’ R: 5’ CACAGACCTGCCGGCTTCGC 3’
*TGFB1*	R: 5’ GGAAATTGAGGGCTTTCGCC 3’ R: 5’ CCGGTAGTGAACCCGTTGAT 3’

### Immunophenotyping

Immunophenotyping of whole blood samples of the patients was performed before and after IMRT and SABR as previously described [22, 23]. Briefly, direct antibody staining of whole blood samples was performed without previous isolation of peripheral blood mononuclear cells. This allows detection of all circulating immune cells including the granulocytic compartment with multicolor flow cytometry and also reduces the required preparation steps. We here used the IPT5 assay with four measuring tubes, focusing on absolute cell count, general immune status, T cells, and dendritic cells in more detail. In sum, 130 immune cell characteristics were measured, including major subsets, further subsets in counts, and activation markers in percentages. The following antibodies conjugated to the indicated fluorochromes were used for the stainings: CD4-PCC5.5, CD45-PECy7, CD45RA-PECy7, CD16 (B73.1)-PE, CD56-FITC, CD11c-V450, CD20-PCC5.5, CD19-PCC5.5, CD28-BB515, CD69-BV421, CD25-PCF594, CD80- PECy7, CD86-PECF594, PD-1-BV421, CD8-FITC, CD19-FITC, CD152-APC, CD197-PE, CD14-FITC, CD3-FITC, CD274-PE, CD20-FITC, CD123-PCC5.5, CD279-APC, CD123-APC, CD16 (3G8)-PE, CD4-APC, CD56- APC-R700 (all BD Biosciences, Heidelberg, Germany); HLA-DR-KO, CD3-KO (all Beckman Coulter, Krefeld, Germany); CD8-PCC5.5, CD14-PCC5.5, CD25-PE-D594, CD86-PED594 (all BioLegend, Biozol, Eching, Germany); CD127-PE-Vio770, HLADR-APC-Vio770 (all Miltenyi Biotec, Bergisch Galdbach, Germany).

### Serum metabolome analysis

Serum samples (10 µL) were analyzed by a targeted quantitative approach using a combined direct flow injection and liquid chromatography (LC) tandem mass spectrometry (MS/MS) assay AbsoluteIDQ 180 kit (Biocrates, Innsbruck, Austria) according to the patented manufacturer’s protocol described in more detail previously [24]. The method combines derivatization and extraction of analytes with selective mass spectrometric detection using multiple reaction monitoring and integrated isotope-labeled internal standards absolute quantification. This strategy allows simultaneous quantification of 185 metabolites: 40 amino acids and biogenic amines, 40 acylcarnitines, 90 glycerophospholipids, and 15 sphingomyelins (Supplementary Table 2). Mass spectrometry analyses were carried out on a TSQ Vantage EMR (Thermo SCIENTIFIC) equipped with a Surveyor HPLC system (Thermo SCIENTIFIC) using an Agilent Zorbax Eclipse XDB-C18 (3.5 μm) 3.0 × 100 mm column and controlled by Xcalibur 2.1. software. The acquired data were processed using Xcalibur 2.1. and MetIDQ (Biocrates Life Sciences AG) software. Concentrations of all metabolites were calculated in μM. To assess the significance of changes between consecutive samples of each patient, the paired *t*-test was employed with *p* < 0.05 as the significance level.

### Human cytokine antibody array analysis

The Proteome Profiler Human XL Cytokine Array Kit (R&D Systems, Lille, France) allows simultaneous quantification of 105 cytokines and inflammatory mediator proteins in one sample. Membranes were hybridized using 200 µL of patient serum according to the manufacturer’s guidelines. The arrays were imaged together using the Chemidoc™ MP imaging system (Bio-Rad). Quantification of signal intensity was performed using ImageLab Version 4.0.1 software (Bio-Rad). The values of duplicate spots representing one protein were averaged, and the background signal was subtracted.

Data for each array were then normalized to the positive control signals to adjust for between-plate effects. Each background-subtracted intensity signal was multiplied by a normalization factor defined as the ratio of the average signal intensity of the positive control spots on all analyzed arrays to the average signal intensity of the positive control spots located on the particular array being normalized. Results are illustrated as mean signal (pixel) intensity for a given protein in each sample. Negative normalized signal intensities were considered as undetected. Due to the very low quality of an array for IMRT sample D (only 20% of antibodies detected), all arrays for that patient were filtered out from further analysis. Subsequently, antibodies which were detected on at least 28 arrays (93% of all arrays) were chosen for further continuous data analysis, resulting in 48 antibodies to analyze. Data imputation for six missing measurements was performed using the kNN algorithm with k = 1 and Euclidean distance to obtain a balanced design. Finally, the measurements for 48 antibodies on 28 arrays (7 patients) were further analyzed, following the basic pipeline described below.

### Data analysis

#### The basic data analysis pipeline

All statistical analyses were performed on normalized data. Classical statistical inference was supported by the effect size analysis to minimize the impact of small sample size and low power of statistical tests [25, 26]. Effect size, unlike a significance test, is independent of sample size. We applied restrictive thresholds of at least large effect size for reporting differences between subjects under comparison, to minimize the small-sample impact.

In the case of between-therapy comparisons, the Mann–Whitney U test was performed with the Benjamini–Hochberg correction for multiple testing. A Wendt’s rank-biserial coefficient of correlation [27] (denoted as r_U_) was calculated to estimate the effect size.

For time/dose series analysis, the repeated-measures analysis of variance (ANOVA) test for paired observations was performed. Post-hoc analysis was performed using a modified Tukey honest significant difference (HSD) test with correction for paired observations. To quantify the effect size, Cohen’s d for paired observations [25] (further denoted as d) was computed. In the case of immunophenotyping data, a nonparametric Wilcoxon test for paired data was performed with corresponding rank-biserial correlation r_W_ calculation as a measure of effect size [28].

Linear interpolation for IMRT samples was performed to estimate the putative signal values at dose points of 7.25 Gy and 36.25 Gy, which allowed for cross-therapy dose–space unification. Linear regression for known dose points was performed, and the obtained model served as the reference for signal estimation (i.e., in the case of a 7.25 Gy value approximation, a linear model between 0 Gy and 10 Gy measurements was constructed). Subsequent statistical analysis of measurements in common dose points was analogous to the one described above.

#### Effect size interpretation

Cohen’s d effect size values ≥1.2 were interpreted as indicating at least a very large effect [25], and the values ≥0.8 as the evidence for at least a large effect. In the case of effect size measured by rank-biserial coefficients of correlation (both r_U_ and r_W_), the critical value for at least a large effect was set to 0.5. The effect size quantification was done for the absolute values of the relevant statistics. Large and very large effect size values indicate differences with a very high level of confidence. For example, an at least large effect size—Cohen’s d ≥ 0.8—implies that at least 79% of observations from one group will have a higher value than the mean of the second group.

Data analysis and visualization was performed in R [29].

## Results

### Gene expression profile of stress-responsive and cytokine genes in blood cells during RT

**Fig. 2:**
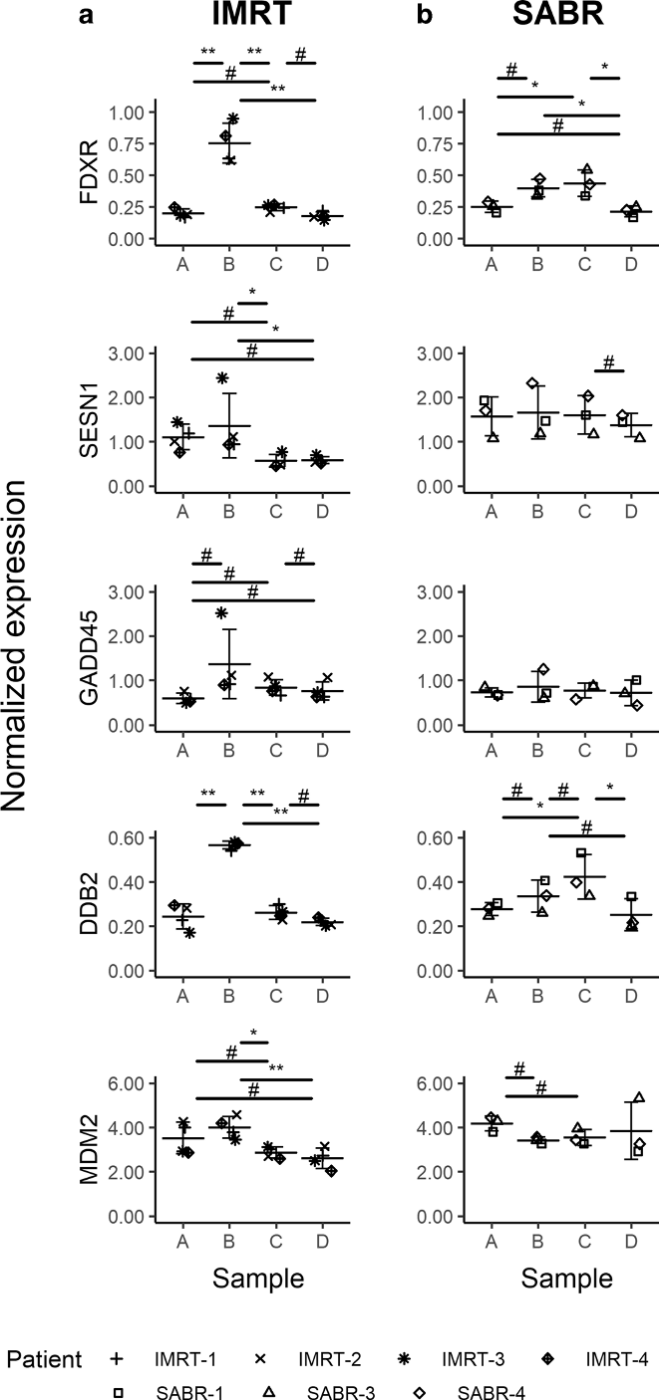
Gene expression of *FXDR*, *SESN1*, *GADD45*, *DDB2*, and *MDM2* in blood of prostate adenocarcinoma patients treated with IMRT (**a**) and SABR (**b**), respectively. Blood was collected before the start of the treatment (blood collection point *A*), after 5 fractions for the IMRT group and after the first for the SABR group (blood collection point *B*), after the last fraction (blood collection point *C*), and 1 month after the last fraction (blood collection point *D*). Data are shown as individual datapoints together with the mean ± SD. Significant *p*-values for the Tukey honest significant difference (HSD) test: *Asterisk* for *p* < 0.05 and *double asterisk* for *p* < 0.01; *hash* indicates a very large effect size (|d| ≥ 1.2). All comparisons found significant by the Tukey HSD test also show large or very large effect sizes, which is not shown on the graph for reasons of clarity

First, the expression profiles of five key radiation-responsive genes (*FDXR, SESN1, GADD45, DDB2*, and *MDM2*) during the course of RT in the blood of 8 prostate cancer patients (Table 1) treated with IMRT (Fig. 2a) and SABR (Fig. 2b) were analyzed. The expression of *FDXR* and *DDB2* was found to increase in both RT treatment modalities (Fig. 2). These two genes were upregulated after the first five fractions (sample B, Fig. 2a) in patients treated with IMRT and their expression went back to background levels at the end of the therapy. The expression of *FDXR* and *DDB2* was also upregulated in SABR patients, but here most pronounced in sample C, i.e., at the end of the treatment (Fig. 2b). In contrast, *SESN1, MDM2*, and *GADD45* expression levels were only slightly altered during the course of the treatment when compared to the pretherapy samples (Fig. 2).

**Fig. 3:**
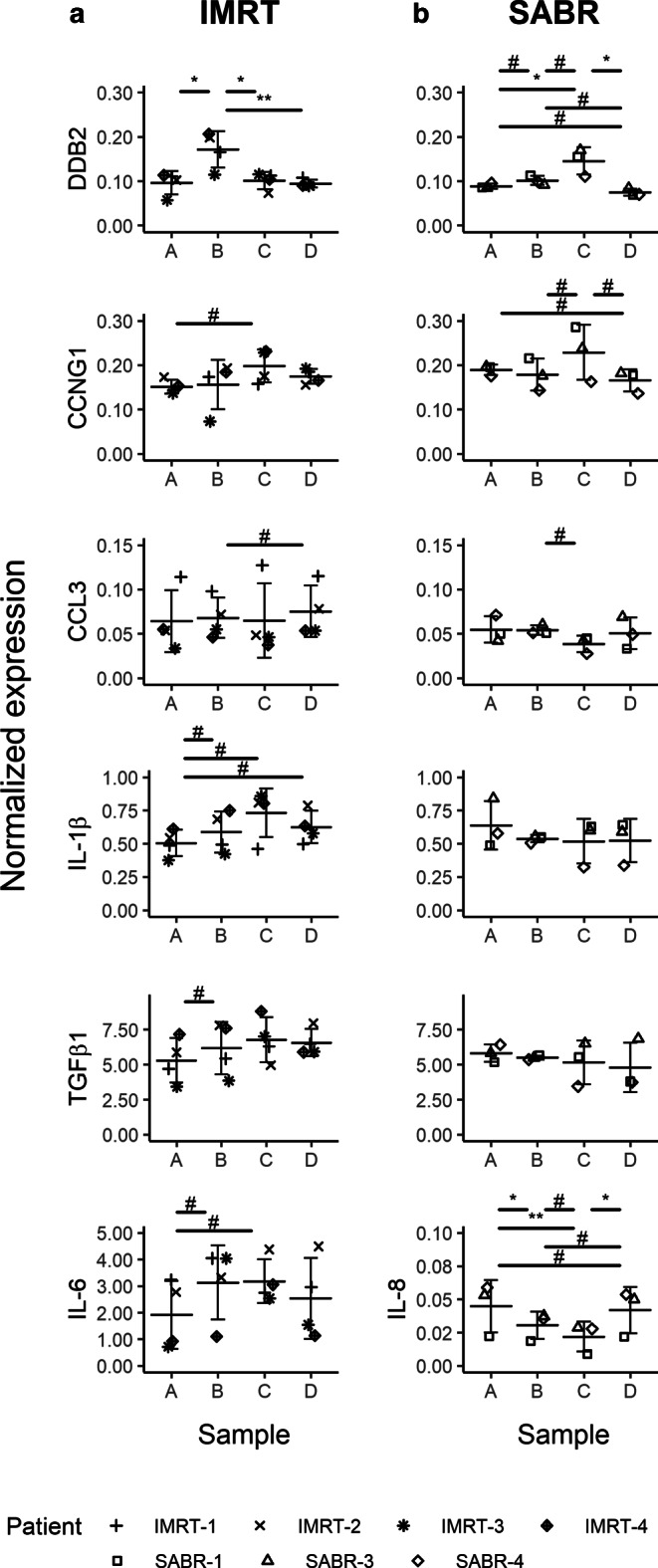
Gene expression of *CCNG1*, *DDB2*, *CCL3*, *IL1B*, *TGFB1*, and *IL6* or *IL8* in blood of the prostate adenocarcinoma patients treated with IMRT (**a**) and SABR (**b**). Blood was collected before the start of the treatment (blood collection point *A*), after 5 fractions for the IMRT group and after the first for the SABR group (blood collection point *B*), after the last fraction (blood collection point *C*), and 1 month after the last fraction (blood collection point *D*). Data are shown as individual datapoints together with the mean ± SD. Significant *p*-values for Tukey honest significant difference (HSD) test: *asterisk* for *p* < 0.05 and *double asterisk* for *p* < 0.01; *hash* indicates a very large effect size (|d| ≥ 1.2). All comparisons found significant by the Tukey HSD test also show large or very large effect size, which is not shown on the graph for reasons of clarity

Additionally, modulation of the expression of a different set of selected genes from the same RNA samples but with a different RT-qPCR assay was investigated. First, to validate radiation-induced changes in the expression of the target genes of interest with a more classical SyBr Green qPCR assay, the expression of *DDB2* was reanalyzed in this experimental system. A similar expression profile to that obtained with the multiplex TaqMan assay for *DDB2 *in IMRT and SABR patients was observed (Fig. 3a, b), i.e., a peak of expression in fractions B and C, respectively (*p* < 0.05 and effect size values |d| > 1.8). Following this validation, the expression of genes coding for a selection of cytokines was analyzed in these samples, together with the gene coding for cyclin G1 as a second stress-responsive gene. *CCNG1* expression was found to be significantly higher in sample C when compared to samples B and D (Fig. 3b) in SABR patients (|d| > 2.1) but not in IMRT patients (Fig. 3a).

Expression of the *TGFB1* gene, coding for a cytokine with anti-inflammatory properties, was found to be increased in IMRT patients when compared to pre-RT levels. This modulation was not observed in SABR patients. Similarly, expression of the pro-inflammatory cytokine *IL1B* gene was induced during the course of treatment and stayed high in the following weeks when compared to the pretherapy sample, but only in IMRT patients (d < −1.4 for all pairwise comparisons). In contrast, a decrease in expression of the gene coding for the C‑C motif chemokine ligand 3 (*CCL3*), an inflammatory factor involved in recruitment and activation of granulocytes, during RT between samples B and C was observed only in SABR patients. Expression of *IL6*, coding for a pro-inflammatory mediator with multiple activities, and *IL8*, another chemokine involved in neutrophils recruitment, was consistently detected only in IMRT and SABR patients, respectively. The expression of *IL6* was increased in both samples B and C when compared to the pretherapy sample (d < −1.2; Fig. 3a).The expression of *IL8* was reduced after the first fraction (sample A vs. sample B) and during the SABR treatment (sample B vs. sample C). The *IL8* mRNA level then increased again in the weeks following RT completion, but without reaching pre-RT levels (Fig. 3b).

**Fig. 4:**
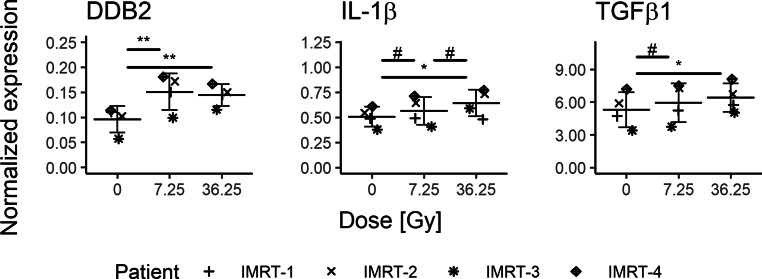
Normalized gene expression values for *DDB2*, *IL1B*, and *TGFB1* gene expression in IMRT patients, interpolated for radiation doses 7.25 Gy and 36.25 Gy. Data are shown as individual datapoints together with the mean ± SD. Significant *p*-values for the Tukey honest significant difference (HSD) test: *asterisk* for *p* < 0.05 and *double asterisk* for *p* < 0.01; *hash* indicates a very large effect size (|d| ≥ 1.2). All comparisons found significant by the Tukey HSD test also show large or very large effect size, which are not shown on the graph for reasons of clarity

However, one should consider that the total dose received and the number of fractions are different in IMRT and SABR patients at samples B and C. These differences may explain the different gene expression patterns observed for, e.g., *DDB2*. To take this into account, gene expression values were approximated for IMRT patients for the radiation doses 7.25 Gy and 36.25 Gy, corresponding to SABR fractions B and C. The expression of *DDB2* was again found to be higher than in the sample before RT, both after 7.25 Gy (*p*-value = 0.001, d = −4.9) and 36.25 Gy (*p*-value = 0.002, d = −6.7) “virtual-doses” (Fig. 4). Importantly, in this interpolation, there is a similar level of expression of *DDB2* in IMRT and SABR patients at the dose of 36.25 Gy (*p*-value = 0.875, r_U_ = 0.2, data not shown), suggesting that the total dose received might be the main parameter driving radiation-induced gene expression. Also, the expression of *TGFB1* was upregulated after 7.5 Gy and 36.25 Gy when compared to pre-RT values (Fig. 4). *IL1B* gene expression was also significantly increased (Fig. 4). Furthermore, effect size statistics suggest that the expression after 36.5 Gy is higher than after 7.25 Gy, even if this effect is not as strong for *TGFB1* (d = −0.9) as for IL-1β (d = −1.4). Altogether, these observations and calculations suggest that the expression of *IL1B* and *TGFB1 *genes is progressively induced during IMRT.

We additionally compared the modulation of inflammatory gene expression in both therapies. Pairwise comparison of the normalized gene expression values in the different samples by a Mann–Whitney U test did not show any significant difference. However, the finding of large effects sizes (|r_U_| ≥ 0.5) between certain pairs of samples suggests that cytokine gene expression is differentially modulated during IMRT and SABR. As shown in Table 3, a higher expression level of *CCL3* and *TGFB1* genes for IMRT patients as compared to SABR patients in samples C and D was observed, while this was not the case in samples A and B (|r_U_| < 0.5). These results indicate that IMRT treatment induces a stronger increase in *CCL3* and *TGFB1* gene expression by the end of the RT compared to SABR. This higher level of expression is maintained in the weeks following RT completion. *IL1B* was found to be more expressed in SABR patients in pre-RT sample A, but this difference was abrogated in sample B (|r_U_| < 0.5). This trend was inverted in sample C, where *IL1B* becomes more expressed in IMRT patients. This difference disappeared in the weeks following RT completion. Thus, the expression of these three genes is clearly more induced during IMRT compared to SABR. The differential upregulation was maintained in the weeks following RT completion for *CCL3* and *TGFB1*, while it was only transitory for *IL1B*.

**Table 3:** Comparison of cytokine gene expression in IMRT and SABR samples A–D

Target	Sample	Mann–Whitney U* p*-value	Wendt’s effect size r_U_
*CCL3*	A	0.857	−0.167
	B	0.629	−0.333
	C	0.229	**−0.667**
	D	0.229	**−0.667**
*IL1B*	A	0.400	**0.500**
	B	1.000	0.000
	C	0.229	**−0.667**
	D	0.857	−0.167
*TGFB1*	A	0.857	0.167
	B	0.857	−0.167
	C	0.400	**−0.500**
	D	0.400	**−0.500**

Values indicating at least large effect size (r_U_) are indicated in bold

### Immunophenotyping of blood cells during RT

**Fig. 5:**
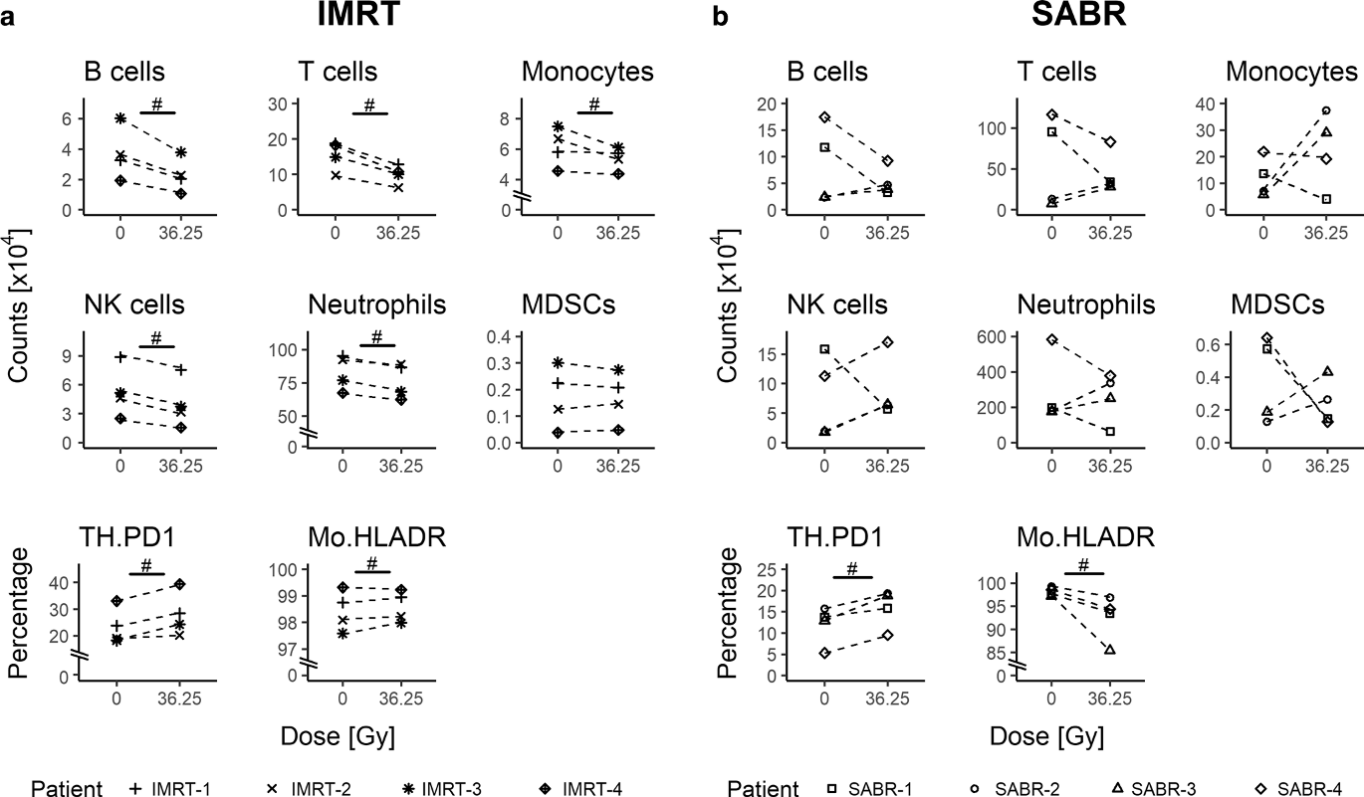
Immunophenotyping of whole blood of the prostate adenocarcinoma patients treated with IMRT (**a**) and SABR (**b**). Whole blood was collected before the start of the treatment and after the last radiation fraction. Expression values were interpolated for IMRT patients for radiation dose of 36.25 Gy for better comparison with SABR-related values. The counts of B cells, T cells, neutrophils, natural killer (*NK*) cells, monocytes, and myeloid-derived suppressor cells (*MDSCs*) per ml of peripheral blood are displayed as well as the percentages of HLA-DR-expressing monocytes (*Mo*) and programmed death 1 receptor-expressingT helper cells (TH.PD1). *Hash* indicates comparisons showing at least a large effect size (|r_W_| ≥ 0.5)

Besides changes in expression of stress-responsive and cytokine genes in blood cells during RT, general cellular immune modulations might take place. Therefore, immunophenotyping of whole blood of the patients was performed. As expected for a systemic immunological response after RT, a decrease of B cells, T cells, and NK cells in the peripheral blood was observed following IMRT. However, such a decline was only observed in 2/4 patients following SABR, and in 2 patients, the neutrophils and monocytes increased following SABR. However, regarding immune-suppressive myeloid-derived suppressor cells (MDSCs), a high increase was only observed in patients who were treated with SABR. Interestingly, again only after SABR, a decrease of HLA-DR expression on monocytes was detected in all patients. One common feature of both treatments was an increased expression of the inhibitory programmed death 1 (PD-1) receptor on CD4+ T cells (Fig. 5).

### Changes in metabolic products during RT

**Fig. 6:**
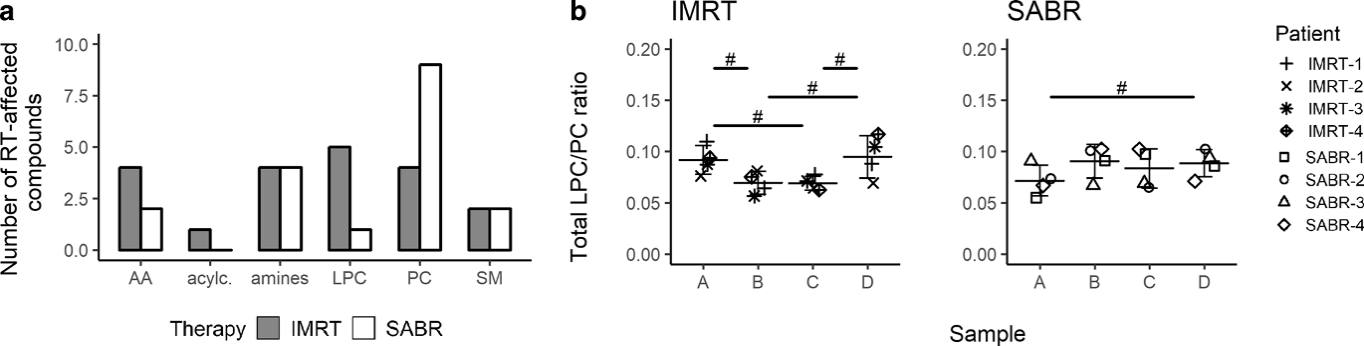
Radiotherapy-related changes in serum metabolome of the prostate adenocarcinoma patients treated with IMRT and SABR. **a** Numbers of compounds in different classes of metabolites that changed their abundances between pre-RT sample A and either post-RT sample B, C, or D (uncorrected *p* < 0.05). Samples from patients subjected to IMRT and SABR are compared in the following classes: phosphatidylcholines (*PC*; 67 compounds in total), lysophosphatidylcholines (*LPC*; 9), sphingomyelins (*SM*; 13), acylcarnitines (*acylc*.; 11), amino acids (*AA*; 21), biogenic amines (*amines*; 16). **b** Changes in the ratio of total LPC versus PC (*LPC/PC*) quantitated in sera of individual patients from both groups. *Hash* indicates comparisons showing at least a very large effect size (|d| ≥ 1.2)

To obtain further deeper insights in distinct systemic modulations following IMRT and SABR, 137 metabolites were quantified in sera of the cancer patients (listed in Supplementary Table 2). Differences in the abundance of selected compounds were observed in individual patients, but few remained statistically significant due to the small size of the patient group (uncorrected *p*-value <0.05). However, marked differences between IMRT and SABR patients were noted for two classes of metabolites: phosphatidylcholines (PCs) and lysophosphatidylcholines (LPCs). There were more PCs affected by RT in the IMRT group than in the SABR group (in general, 18% of detected PCs were RT affected in either group and sample). On the other hand, there were more LPCs affected by RT in the SABR group than in the IMRT group (in general, 56% of detected LPCs were RT affected; Fig. 6a). Therefore, changes in the total LPC/PC ratio in samples of individual patients were analyzed (Fig. 6b). In the SABR group, this ratio generally increased in sample B (that corresponded to the effect of a single 7.25 Gy dose), with a large effect size (d = −0.9). On the other hand, in the IMRT group, this ratio generally decreased in sample B (that corresponded to the effect of five 2 Gy fractions), with a very large effect size (d = 1.5). In both cases, changes in the total LPC/PC ratio resulted from changes in levels of both LPCs and PCs. Importantly, a pairwise comparison of both therapies revealed a significant difference (effect size r_U_ = 0.75) in individual changes between sample A and sample B. The LPC/LC ratio returned to the pre-RT value in the post-RT sample D that was collected 1 month after the end of treatment in IMRT patients.

### Modulation of cytokines, inflammatory proteins, and immune regulators abundance in plasma during RT

Finally, the modulation of the level of a collection of 105 cytokines and proteins was analyzed in the serum of the cancer patients during the course of RT by using dedicated antibody arrays. This analysis was restricted to a subgroup of 48 proteins which gave a measurable signal in at least 26 of the 28 antibody arrays analyzed (one array/sample for each patient). Out of these 48 cytokines, an ANOVA test for repeated measures showed statistical differences (*p*-value <0.05) in the level of expression at different timepoints for only six factors (Table 4 and Supplementary Fig. 1): BAFF (B-cell activating factor), complement factor D (CFD), C‑reactive protein (CRP), thrombospondin‑1, and dipeptidyl peptidase 4 (DPPIV) for IMRT patients, and the soluble form of the T‑cell immunoglobulin and mucin-domain containing‑3 receptor (Tim-3) for SABR patients.

**Table 4:** Serum proteins showing very large effect sizes between at least three pairwise sample comparisons (out of six possible) during RT

SABR	IMRT
–	**BAFF**
–	Chitinase 3‑like 1
–	**Complement factor D**
–	**C‑reactive protein**
–	**DPPIV**
GDF-15	GDF-15
IGFBP‑2	–
–	IGFBP‑3
–	MMP‑9
–	RANTES
–	Resistin
–	SHBG
–	**Thrombospondin‑1**
–	CD31
**TIM‑3**	TIM‑3
–	VCAM‑1

Proteins for which ANOVA *p*-value was statistically significant (*p* < 0.05) are indicated with bold.

*BAFF* B‑cell activation factor, *CD31* Platelet and endothelial cell adhesion molecule 1, *CRP* C‑reactive protein, *DDPIV* Dipeptidyl peptidase 4, *GDF-15* Growth and differentiation factor 15, *IGFBP* Insulin-like growth factor binding protein, *MMP 9* Matrix metallopeptidase 9, *RANTES* Regulated on activation, normal T cell expressed and secreted, *SHBG* Sex hormone-binding globulin, *Tim‑3* T‑cell immunoglobulin and mucin domain-containing 3, *VCAM‑1* Vascular cell adhesion molecule 1

However, further analysis showed a very large effect size (|d| ≥ 1.2) for the expression of several other soluble factors at different timepoints during RT. To identify those modulated during the course of RT with the highest level of confidence possible in our setting, we selected only those proteins for which at least three of the six possible pairwise comparisons between the four samples resulted in very large effect sizes, indicating differences between at least three samples. This analysis resulted in the identification of an additional group of serum proteins with modulated expression during and/or after RT, as compared to the pre-RT sample (Table 4 and Supplementary Fig. 1). Altogether, with these criteria, 15 out of the 48 proteins detectable on the antibody array membranes were found to be modulated during IMRT, but only 3 during SABR. Only 2 proteins were modulated in both treatments: growth and differentiation factor 15 (GDF-15) and Tim‑3.

Only 3 of the 15 factors modulated during IMRT, namely CRP, GDF-15, and the sex hormone-binding globulin (SHBG) were found to be increased in sample B when compared to pre-RT sample A, whereas four (BAFF, CFD, DDPIV, and resistin) were decreased. The serum level of 10 of these factors (BAFF, chitinase 3‑like 11, CRP, CFD, DPPIV, Insulin-like growth factor binding protein-3 (IGFBP 3), matrix metallopeptides 9 (MMP9), resistin, thrombospondin‑1, Tim-3) was higher in sample C compared to sample B, indicating an increase in their serum concentration during the course of RT. A decrease in the level of several proteins in the weeks following RT completion were also observed. The expression of BAFF, CFD, CRP, GDF-15, IGFBP‑3, SHBG, CD31, and Tim‑3 was lower in sample D vs. sample C. However, for a large subgroup including BAFF, CRP, DPPIV, GDF-15, MMP9, Regulated on activation, normal T cell expressed and secreted (RANTES), thrombospondin‑1, CD31, Tim‑3, and Vascular cell adhesion molecule 1 (VCAM‑1), the level of expression in sample D was higher than in sample A, indicating that the induction of the expression of these cytokines/factors is long lasting and persists at higher levels in the weeks following RT completion compared to the pre-RT samples.

Only three factors were modulated during the course of SABR. GDF-15 and IGFBP‑2 already increased after the first fraction (sample B) and stayed elevated at the end of RT (sample C) when compared to pre-RT levels (sample A). Whereas the GDF-15 level decreased in the weeks following RT completion, the level of IGFBP‑2 stayed elevated. The pattern of modulation of Tim‑3 was clearly different, as its expression was elevated only in sample D, i.e., weeks after RT completion (Supplementary Fig. 1).

**Fig. 7:**
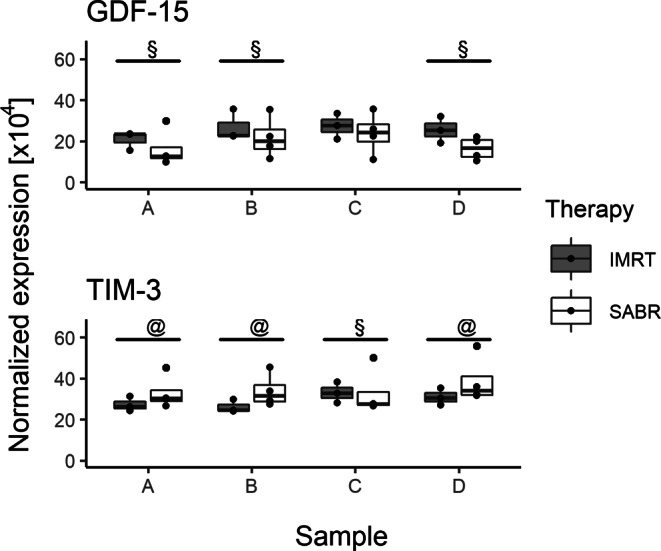
Comparison of the modulation of GDF15 and TIM‑3 proteins during IMRT and SABR. At least large effect sizes (|r_U_| ≥ 0.5) were indicated with an *@* and a *§* sign for positive and negative r_U_ values, respectively

As GDF-15 and Tim‑3 were both found to be regulated during the course of the two RT modalities, we next compared their modulation patterns (Fig. 7). A large effect size (|r_U_| > 0.5) was observed for most of the timepoints. Tim‑3 was expressed more highly in SABR compared to IMRT patient samples A, B, and D, but more expressed in IMRT than SABR patient samples at timepoint C. This pattern suggests that IMRT was able to evoke a stronger induction of Tim‑3 expression than SABR during the RT procedure. This induction subsided in the weeks following the end of the treatment. For GDF-15, a mirrored pattern was observed: GDF-15 was more highly expressed in IMRT patient samples A, B, and D, but not in sample C. Thus, here, it appears that SABR induced the serum level of GDF-15 to a greater extent than IMRT.

## Discussion

This exploratory study presents for the first time a detailed analysis of several stress and immune parameters at a systemic level that might be modulated in vivo following local exposure to ionizing radiation in a small group of patients treated for prostate cancer by RT using IMRT and SABR. Both RT modalities have similar anti-tumor efficiency [30] and a low level of acute toxicity was observed in our patients: only one of the IMRT patients exhibited grade 2 gastrointestinal toxicity. Therefore, the observed modulations of immune or immune-related parameters reported in this study can be considered to reflect the effects of locally applied ionizing radiation on systemic immune alterations in patients with prostate adenocarcinoma rather than differences in radiation-induced toxicity.

Overall, the exposure of IMRT and SABR patients differed in terms of the total dose delivered over the RT course, the dose per fraction, the number of fractions, the dose rate, and the irradiated CTV. Patients treated with IMRT and one patient treated with SABR received androgen-deprivation therapy. ADT has been shown to induce a rapid recruitment of T lymphocytes and antigen-presenting cells to the tumor bed [31]. ADT can also induce a rebound in the production of naïve peripheral T lymphocytes in prostate cancer patients [32]. However, no effects of ADT on the homeostasis of B, T, and NK cell subsets in the peripheral blood of treated patients were observed in a comparison of healthy donors and treated patients [33]. In this study, immunophenotyping did not reveal any specific modulation of lymphocyte populations in ADT-treated patients before or during RT. One can therefore conclude that ADT did not interfere with the analyses of systemic modulations during IMRT and SABR. The observed differences should rather result from differences in radiation volume and/or dose and/or treatment technique, including the number of fractions delivered. Because of the small size of 4 patients per treatment group, putative differences between RT modalities might be masked by inter-individual differences between patients. To circumvent this potential problem, we compared the evolution of the modulation of the different endpoints during IMRT and SABR, and not their absolute level. Furthermore, effect size values were calculated. In contrast to statistical testing, effect size does not depend on sample size and thus can provide more reliable results. The use of a restrictive threshold (at least a large effect size) allowed identification of the strongest differences and variations between samples with a high level of confidence.

In this study, a large array of stress and immune parameters at the systemic level in the blood of IMRT- and SABR-treated prostate cancer patients was analyzed. The average total dose delivered to the patients’ blood during RT in the treating center (with an estimation based on the individual treatment plans, assuming that the blood volume corresponds to 8% of the irradiated body mass and that there is a homogenous vascularization of irradiated tissues) was 0.035 Gy per 100 mL and 0.009 Gy per 100 mL in the case of IMRT and SABR patients, respectively. Because of the specificities of IMRT and SABR, samples B and C correspond to different treatment doses and durations. In an attempt to directly compare the effects of these two treatment modalities for the same dose, we interpolated values obtained in IMRT patients in samples B and C to equivalent radiation doses (i.e., 7.25 and 36.25 Gy).

A large body of work has been devoted to the identification of biomarkers of radiation exposure. The level of transcription of selected p53-dependent genes was shown to be a good candidate for both total and partial body exposure over a wide range of ionizing radiation doses [34]. Data on the systemic effects of IMRT and SABR are, however, still scarce. Our analyses revealed a transient increase in the expression of *FDXR* and *DDB2* in both IMRT and SABR patients during the course of RT, with, however, different kinetics. These different kinetics might be related to the total dose delivered at each specific blood collection timepoint and/or to the exposed volume. Interpolating the putative expression levels obtained in IMRT patients to similar doses delivered in SABR suggests that *DDB2* transcription is induced in both treatments already after 7.25 Gy. It further increases until a cumulative dose of 36.25 Gy, suggesting similar induction mechanisms during the course of SABR and IMRT treatments.

In contrast, the effects of local radiation exposure on immune parameters at the cellular and humoral levels were different in patients treated with IMRT and SABR. The induction of transcription of *IL1B* and *TGFB1* genes took place only in IMRT patients. Generally, IMRT resulted in modulation of a much larger number of cytokines and proteins in the serum compared to SABR. Evolution of the LPC/PC ratio, a postulated metabolomic indicator of inflammation [19, 20], was strikingly different during the course of IMRT and SABR. Altogether, these observations suggest that inflammatory mechanisms are differentially regulated during the course of IMRT and SABR. In contrast, the effects of RT on the expression of certain activation markers in circulating immune cell subsets was very homogeneous. In all patients, an increase in the percentage of PD-1-expressing CD4^+^ T lymphocytes was for example observed at the end of the treatment.

The impact of even low doses of ionizing radiation on the expression of activation markers by immune cell subsets has already been observed [9]. Modulation of anti-tumor immune responses by interfering with PD-L1/PD‑1 interactions has systemic effects on the T cell compartment. Thus, an increase in circulating PD‑1^+^ CD4^+^ T cells (and in myeloid-derived suppressor cells) following RT completion was, for example, associated with reduced T cell activity in colorectal cancer patients [35]. However, on the other hand, RT can also result in an increased frequency of circulating tumor-specific T cells in the CD8^+^ population, as observed in colorectal and prostate cancer patients [36], and the frequency of circulating HLA-DR^hi^ monocytes is a strong predictor of progression-free and overall survival in response to therapy in stage IV melanoma patients [37]. It was already described in 1999 that even though RT reduces immune cell numbers in the peripheral blood, the remaining lymphocyte function was still within the normal range [38]. Our findings confirm such immune modulations in prostate adenocarcinoma patients. In addition, the results of this explorative study provide—for the first time—indications that different RT protocols and application methods such as IMRT and SABR modulate several components of the immune system differently, as well as their dynamics. Interestingly, in patients treated with IMRT, changes in the level of BAFF, a survival factor of B cells, correlated with absolute numbers of B cells. Thus, local radiation exposure partially exerts its systemic effects by altering immune cell subsets, their activation status, and also their microenvironment.

The immunological consequences of DNA damage are becoming more and more evident [39]. Interestingly, during IMRT, expression of the *DDB2* gene appears to be already increased after 7.25 Gy, whereas induction of the expression of *IL1B* and *TGFB1* genes in the same samples is only evident after 36.25 Gy. Similarly, the serum concentration of the inflammatory cytokines and proteins modulated during IMRT was mostly upregulated only between samples B and C, but not in sample B compared to the pre-RT sample A. This delay in the induction of stress-response genes and of inflammatory parameters in the peripheral blood cells suggests that these events are differentially regulated in blood cells with individual dynamics. It may be that inflammatory genes are not directly induced by radiation, but rather that their up-regulation is a consequence of the biological effects elicited by radiation exposure, namely tumor cell death and the death of at least a fraction of irradiated circulating blood cells [40]. Irrespective of their identity, these mechanisms and their outcome(s) are complex, as shown by the findings that both IL-1β, a prototypic pro-inflammatory cytokine, and TGFβ1, a prototypic anti-inflammatory cytokine, were coregulated during IMRT. Another example is the coregulation of the chemokine RANTES and of the exopeptidase DDPIV, as proteolytic cleavage of RANTES by DDPIV can modulate its activity. The simultaneous upregulation of effectors with opposing activities indicates that radiation-induced stress and immune effects are multifaceted. The balance of these activities is finely regulated during the responses elicited by IMRT and SABR. In the rare cases of a similar factor being modulated in patients treated with either IMRT or SABR, this modulation appears to be in opposite directions in the different RT modalities, as shown in our study of GDF-15 and Tim‑3. GDF-15 is a member of the TGFβ family with both pro- and anti-tumor activities [41]. Its expression is associated with cellular stress conditions like irradiation in human PBLs [42], primary fibroblasts exposed ex vivo [43, 44], and murine splenocytes following whole-body exposure [45]. Our results now show that it is also transiently induced in vivo during RT, with specific regulation in each RT modality. This difference may be linked to the different initial levels of GDF-15 in both groups of patients, or to differences in cell death/stress or tissue damage. We did indeed observe regulation of several factors denoting endothelial dysfunction (CD31, VCAM, thrombospondin) and tissue remodeling (MMP9, chitinase 3‑like 1) in IMRT, but not in SABR patients. The other differently regulated protein common to both groups of patients is Tim‑3, a negative regulator of Th lymphocytes and innate immune cell activation when expressed on the cell surface [46]. In vitro, Tim‑3 has been found to be shed by ADAM proteases from the surface of human TLR-activated CD14^+^ monocytes [47, 48]. Here, we observed a low but detectable persistent increase in the circulating level of Tim‑3 in the weeks following the end of the treatment in both IMRT and SABR patients. So far, no function has been attributed to this soluble Tim‑3 protein, but its increase may reflect innate immune cell activation during IMRT and SABR treatments.

Even though this explorative study was already quite comprehensive, future studies should be even more detailed and include more immune parameters such as danger signals that reflect radiation-induced immune damage response [49] or HSP70 abundance [50], which give indications about the tumor status.

In addition, future studies should also consider additional endpoints such as DNA damage, including complex and oxidative lesions, apoptosis, the use of sensitive methods such as enzyme-linked immunosorbent assay (ELISA) to measure the modulation of circulating inflammatory cytokines, and of course clinical outcome. The level of a collection of cytokines was found to be differentially regulated in lung cancer patients treated by RT only vs. patients treated with RT + chemotherapy, and the early modulation of some of these cytokines was correlated with lung toxicity [15]. It was also shown that DNA damage could be detected in non-irradiated, out-of-field tissues in a different cohort of patients treated by RT or RT + chemotherapy for lung cancer [51]. This abscopal effect is similarly observed in pre-clinical mouse models locally exposed to a single dose of high-dose-rate synchrotron radiation [52, 53]. In mice, the generation of genotoxic lesions at distant sites was found to depend on a functional immune system and was attenuated in the absence of the CCL2/MCP‑1 cytokine. It would be interesting to find out whether the different modulation of immune parameters that we observe in IMRT and SABR patients translates into a differential pattern of genotoxic stress at distant sites according to RT modality in patients. Pre-clinical mouse studies could be designed to investigate the eventual relations between dose, dose rate, repeated exposure, immune modulation, and genotoxic effects at distant sites in the context of local irradiation of normal tissue or tumors.

In conclusion, the data presented herein depict that localized irradiation results in systemic modulation of a large range of cellular and humoral immune parameters. The radiation-induced effects on inflammatory gene transcription, immune cell homeostasis, and serum concentration of lipids and cytokines appear to be different in patients treated by different RT modalities. These differences are qualitative (e.g., genes induced only in IMRT, more cytokines modulated in IMRT, myeloid cells modulated only in SABR) and quantitative (e.g., different evolution of LPC/PC ratio). This exploratory study suggests that RT modalities with similarly high therapeutic efficiency and low clinical toxicity can have different effects on immune system homeostasis and activation at the systemic cellular and molecular level for up to 5 weeks following RT completion. Late effects of RT such as a decrease in circulating CD3^+^ T lymphocytes have previously been observed in prostate cancer patients 12 months post treatment [54]. Thus, the findings presented here need to be confirmed in larger groups of patients in the future, also taking into account further analyses at even later timepoints after completion of RT.

## Caption Electronic Supplementary Material

Supplementary Table 1: Full description of the patients recruited for this study

Supplementary Table 2: Metabolites quantified in the serum of PAC patients

Supplemntary Figure 1: Representation of the modulation of serum proteins listed in Table 4 during RT.

## Abbreviations

ADT: Androgen deprivation therapy
BAFF: B‑cell activation factor
CCNG1: Cyclin G1
CD31: Platelet and endothelial cell adhesion molecule 1
CRP: C‑reactive protein
DDB2: Damage-specific DNA binding protein 2
DDPIV: Dipeptidyl peptidase 4
FDXR: Ferredoxin reductase
GADD45: Growth arrest and DNA damage inducible
GDF-15: Growth and differentiation factor 15
IGFBP: Insulin-like growth factor binding protein
IL: Interleukin
IMRT: Intensity-modulated radiation therapy
LPC: Lysophosphatidylcholine
MDM2: Mouse double minute 2 homolog
MMP 9: Matrix metallopeptidase 9
PBLs: Peripheral blood lymphocytes
PC: Phosphatidyl choline
PD‑1: Programmed death receptor 1
PD-L1: Programmed death receptor ligand 1
RANTES: Regulated on activation, normal T cell expressed and secreted
RT: Radiotherapy
SABR: Stereotactic ablative body radiotherapy
SESN1: Sestrin 1
SHBG: Sex hormone-binding globulin
Tim‑3: T‑cell immunoglobulin and mucin domain-containing 3
TLR: Toll-like receptor
VCAM‑1: Vascular cell adhesion molecule 1
