# Supplementary material for: Systemic modulation of stress and immune parameters in patients treated for prostate adenocarcinoma by intensity-modulated radiation therapy or stereotactic ablative body radiotherapy
Source: Strahlenther Onkol. 2020 Jun 9;196(11):1018–33. doi: 10.1007/s00066-020-01637-5 (PMC7581573; doi:10.1007/s00066-020-01637-5)
Supplement: Supplementary file 2 — Supplementary Table 2: Metabolites quantified in the serum of PAC patients [file 66_2020_1637_MOESM2_ESM.docx]

**Supplemantary Table 2: Metabolites quantified in the serum of PAC patients**

| **Acylcarnitines** | C0; C10; C10:1; C12; C12:1; C14:1; C16; C18:1; C2; C3; C8. |
| --- | --- |
| **Aminoacids** | Ala; Arg; Asn; Asp; Cit; Gln; Glu; Gly; His; Ile; Leu; Lys; Met; Orn; Phe; Pro; Ser; Thr; Trp; Tyr; Val. |
| **Biogenic amines** | ADMA; alpha-AAA; c4-OH-Pro; Creatinine; DOPA; Dopamine; Histamine; Kynurenine; Met-SO; Putrescine; SDMA; Serotonin; Spermidine; Spermine; t4-OH-Pro; Taurine. |
| **Glycerophospholipids** | lysoPC a C14:0; lysoPC a C16:0; lysoPC a C16:1; lysoPC a C17:0; lysoPC a C18:0; lysoPC a C18:1; lysoPC a C18:2; lysoPC a C20:3; lysoPC a C20:4; PC aa C26:0; PC aa C28:1; PC aa C30:0; PC aa C32:0; PC aa C32:1; PC aa C32:2; PC aa C32:3; PC aa C34:1; PC aa C34:2; PC aa C34:3; PC aa C34:4; PC aa C36:0; PC aa C36:1; PC aa C36:2; PC aa C36:3; PC aa C36:4; PC aa C36:5; PC aa C36:6; PC aa C38:0; PC aa C38:3; PC aa C38:4; PC aa C38:5; PC aa C38:6; PC aa C40:1; PC aa C40:2; PC aa C40:3; PC aa C40:4; PC aa C40:5; PC aa C40:6; PC aa C42:0; PC aa C42:6; PC ae C30:0; PC ae C30:1; PC ae C32:1; PC ae C32:2; PC ae C34:0; PC ae C34:1; PC ae C34:2; PC ae C34:3; PC ae C36:0; PC ae C36:1; PC ae C36:2; PC ae C36:3; PC ae C36:4; PC ae C36:5; PC ae C38:0; PC ae C38:1; PC ae C38:2; PC ae C38:3; PC ae C38:4; PC ae C38:5; PC ae C38:6; PC ae C40:1; PC ae C40:2; PC ae C40:3; PC ae C40:4; PC ae C40:5; PC ae C40:6; PC ae C42:0; PC ae C42:1; PC ae C42:2; PC ae C42:3; PC ae C42:4; PC ae C42:5; PC ae C44:4; PC ae C44:5; PC ae C44:6. |
| **Sphingolipids** | SM (OH) C14:1; SM (OH) C16:1; SM (OH) C22:1; SM (OH) C22:2; SM (OH) C24:1; SM C16:0; SM C16:1; SM C18:0; SM C18:1; SM C20:2; SM C24:0; SM C24:1; SM C26:1. |
