# Supplementary figures and images for: Systemic modulation of stress and immune parameters in patients treated for prostate adenocarcinoma by intensity-modulated radiation therapy or stereotactic ablative body radiotherapy
Source: Strahlenther Onkol. 2020 Jun 9;196(11):1018–33. doi: 10.1007/s00066-020-01637-5 (PMC7581573; doi:10.1007/s00066-020-01637-5)

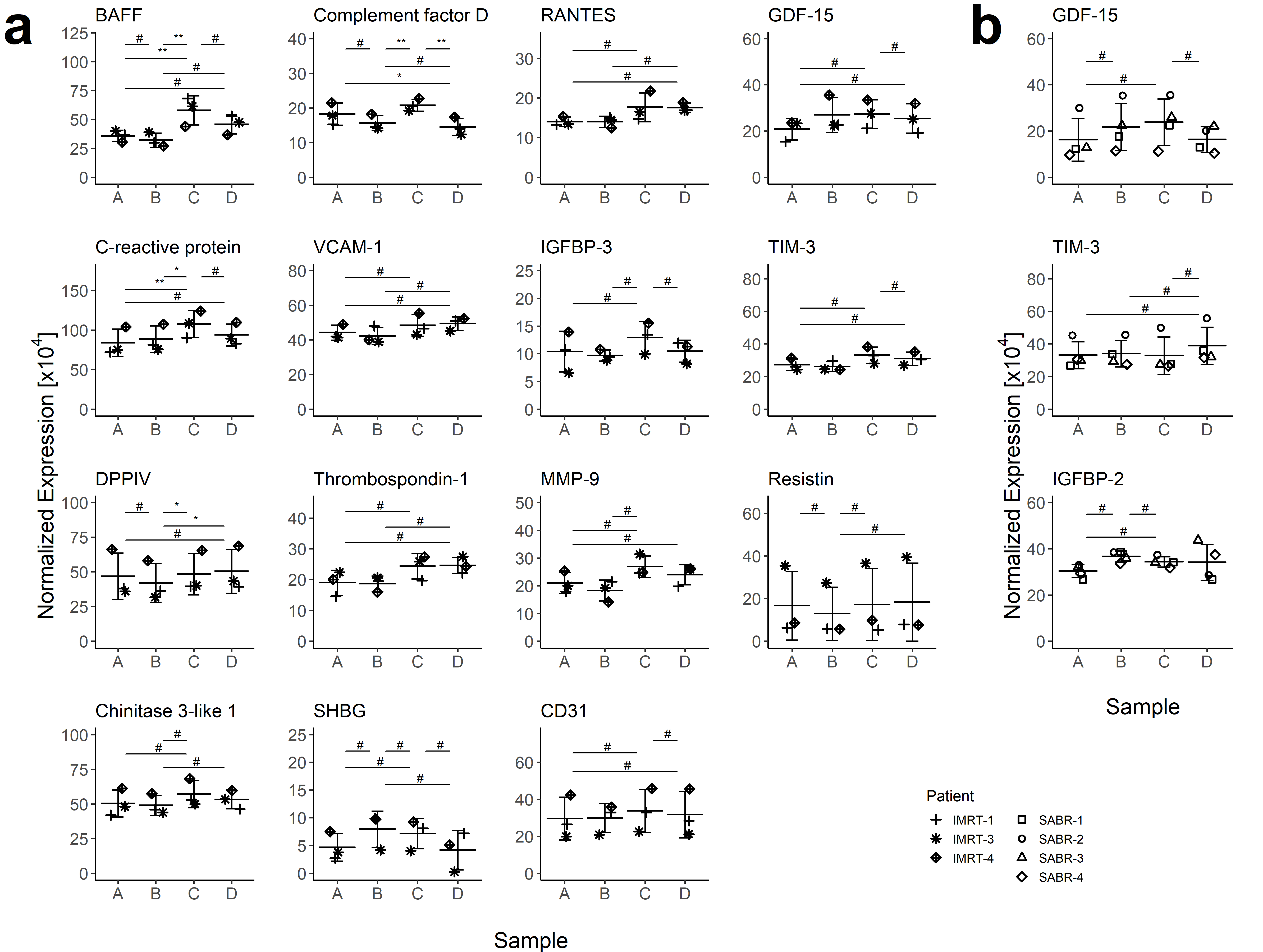

Supplement: Supplementary file 3 — Supplemntary Figure 1: Representation of the modulation of serum proteins listed in Table 4 during RT. [file 66_2020_1637_MOESM3_ESM.tif]
